# ZhenQi FuZheng formula inhibits the growth of colorectal tumors by modulating intestinal microflora-mediated immune function

**DOI:** 10.18632/aging.204111

**Published:** 2022-06-08

**Authors:** Weiqi Meng, Zhiping Li, Yiting Zhang, Anhui Yang, Yanzhen Wang, Yulin Zhou, Wanyue Wu, Ye Qiu, Lanzhou Li

**Affiliations:** 1 Department of Clinical Pharmacy, The First Hospital of Jilin University, Jilin University, Changchun, Jilin, P.R. China; 2 School of Life Sciences, Jilin University, Changchun, Jilin, P.R. China; 3 Department of Pharmacy, Changchun University of Chinese Medicine, Changchun, Jilin, P.R. China; 4 School of Pharmacy and Food Science, Zhuhai College of Science and Technology, Zhuhai, P.R. China; 5 Engineering Research Center of Chinese Ministry of Education for Edible and Medicinal Fungi, Jilin Agricultural University, Changchun, Jilin, P.R. China

**Keywords:** Zhenqi Fuzheng formula, colorectal cancer, intestinal microflora, T cells, immunomodulatory

## Abstract

Zhenqi Fuzheng formula (ZQFZ), of which the main ingredients are *Astragalus membranaceus* and *Ligustrum lucidum*, has immune system regulatory functions and potential anti-tumor bioactivity. The inhibition of colorectal tumor growth by ZQFZ was analyzed in inflammatory cells and B6/JGpt-*Apc*^em1Cin(MinC)^/Gpt (*Apc*^Min/+^) mice. ZQFZ exhibited anti-inflammatory activity by decreasing the phosphorylation of nuclear factor-kappa B (NF-κB) pathway-related proteins in lipopolysaccharide-induced RAW264.7 cells. After 56 days of treatment, ZQFZ alleviated the progression of colorectal cancer (CRC) and increased the body weight and thymic index values of the *Apc*^Min/+^ mice. An analysis of the intestinal microflora showed that ZQFZ affected the abundance of certain immune-related bacteria, which may explain its immunomodulatory effects. Moreover, the percentages of T cells and NK cells in peripheral blood were significantly increased and 15 immune-related cytokines were regulated in serum or the colon or both. ZQFZ upregulated the levels of CD4 and CD8 in the spleen and colorectal tumors and decreased the expression levels of cytotoxic T-lymphocyte-associated protein 4 and programmed death-ligand 1 in colorectal tumors. ZQFZ promoted an anti-tumor immune response and inhibited the occurrence and development of CRC by regulating the immune system. This study provides the experimental basis for the application of ZQFZ as a therapeutic agent for CRC.

## INTRODUCTION

Colorectal cancer (CRC) is the fourth most common cancer globally. Its incidence is only lower than prostate cancer in men, breast cancer in women and lung cancer in the US [1]. CRC accounted for 10.2% and 9.2% of the global cancer incidence and cancer-related mortality, respectively, until 2018, and these percentages are continuously increasing [2]. Based on its risk factors and causes, CRC can be divided into three categories: sporadic CRC (88–94%), CRC associated with inflammatory bowel disease (1–2%) and hereditary CRC (5–10%) [3]. Epidemiological studies have shown that many factors affect the occurrence of CRC by decreasing central adiposity, influencing sexual and metabolic hormone levels, reducing inflammation and improving immune function [4].

Spontaneous CRC is related to older age, environmental factors, a personal history of sporadic tumors and familial CRC. Meanwhile, CRC associated with inflammatory bowel disease originates from ulcerative colitis and Crohn’s colitis [5]. Hereditary nonpolyposis CRC and familial adenomatous polyposis, which are induced by the mutation of adenomatous polyposis coli [6] and mismatch repair genes [7], respectively, are the main forms of hereditary CRC.

In contrast to colitis-associated cancer, inflammation always follows tumorigenesis in CRC. When mutations caused by environmental factors initiate tumor development in CRC, the subsequent activation of inflammatory cells induces further DNA damage through the production of reactive oxygen species and reactive nitrogen intermediates [8, 9]. Immune mediators, such as interleukin (IL)-1β, tumor necrosis factor (TNF)-α and IL-6, which are stimulated by the innate immune sensors Toll-like receptors (TLRs), have been shown to maintain gut homeostasis and are implicated in cell survival, the immune response and inflammation. The production of these mediators is mediated by several major signaling pathways, such as the nuclear factor-kappa B (NF-κB) and signal transducer and activator of transcription 3 pathways [10].

The gut microbiota creates an excellent microecological environment for the host, and it is closely related to the health of organisms and the occurrence and development of diseases [11]. The number of microbial species in the adult human intestine is estimated to exceed 2,000 and the total number of microbes may reach 100 trillion [12]. The gut microbiota constantly communicates with the host, accelerates the maturity of the immune system and metabolizes indigestible ingredients from food [13, 14]. The relationship between the gut microbiota and diseases has become one of the most exciting frontiers in health in the past decade. CRC patients have a higher relative abundance of Bacteroides fragilis, Enterococcus and Escherichia/Shigella, but lower microbiota diversity and a lower abundance of Clostridia [15] than healthy individuals. These intestinal microbes affect the development of CRC via effects on host metabolism, immune function, host/microbial sensing pathways and cellular proliferation [16].

Treatments for CRC currently include surgery, radiotherapy, radio-chemotherapy, chemotherapy and immunotherapy. Surgery has been the standard treatment for patients with non-metastasized CRC over the past few decades, as operative technologies are constantly developing and becoming more accurate [17, 18]. However, the high cost of surgery, poor prognosis, recurrence and the decrease in quality of life caused by subsequent radiotherapy remain as obstacles to effective tumor treatment. Therefore, alternative therapeutic agents as adjuncts to surgery, but with fewer adverse effects and lower costs, are urgently needed. Zhenqi Fuzheng formula (ZQFZ) is a Chinese patent medicine, the classical formulation is a (1: 2, w/w) mixture of Ligustri Lucidi Fructus (dried ripe fruit of *Ligustrum lucidum*) and Astragali Radix (dried root of *Astragalus membranaceus*) [19]. ZQFZ is commonly used clinically to improve immunity and, in combination with chemotherapy, to treat cancers [20]. In our previous study, we demonstrated that ZQFZ has beneficial effects on the hematopoietic system in cyclophosphamide-injected mice. Thus, we propose that ZQFZ may be used as both an immunomodulator and an anti-myelosuppressive agent [21]. A Bayesian network meta-analysis has previously been used to assess the comparative effectiveness and safety of ZQFZ in gastric cancer when combined with chemotherapy [20]. However, it remains unknown whether ZQFZ can inhibit tumor growth or whether it has an anti-tumor effect on CRC.

In this study, the anti-CRC effects of ZQFZ were demonstrated in RAW264.7 cells and B6/JGpt-*Apc^em1Cin(MinC)^*/Gpt (*Apc*^Min/+^) mice. These effects were mainly related to the regulation of inflammation, immunity and the intestinal microflora. Our data indicate the potential of ZQFZ as an adjuvant drug for the clinical treatment of CRC.

## MATERIALS AND METHODS

### Cell culture and ZQFZ treatment

The mouse macrophage cell line RAW264.7 (Procell Life Science and Technology Co., Ltd., Hu Bei, China) was cultured in high-glucose Dulbecco’s modified Eagle medium (DMEM) (ThermoFisher Biochemical Products Co., Ltd., Beijing, China), containing 10% fetal bovine serum (FBS; Procell Life Science and Technology Co., Ltd. Hubei, China), 1% penicillin-streptomycin solution (Procell Life Science and Technology Co., Ltd. Hubei, China) and 0.1% plasmocin prophylactic (InvivoGen, San Diego, CA, USA), at 37°C in a humidified incubator with 5% CO_2_ and 95% air. After overnight culture in a 6-well plate (8 × 10^5^ cells/well, 2 mL of medium/well), the cells were divided into four groups: control, model, 0.25 and 0.5 mg/mL ZQFZ. The ZQFZ-treated cells were pre-treated with various concentrations of ZQFZ (provided by Xiuzheng Pharmaceutical Group Co., Ltd.) dissolved in DMEM containing 1% FBS for 4 h. The model and ZQFZ-treatment groups were then incubated for 24 h with 1 μg/mL lipopolysaccharide (LPS; L4391; Sigma-Aldrich, St Louis, MO, USA) dissolved in phosphate-buffered saline (PBS). Control cells were treated with DMEM containing 1% FBS.

### Animal maintenance and ZQFZ treatment

All animal experimental protocols used in this study were approved by the Animal Ethics Committee of Jilin University (No. SY201910004). Sixteen male *Apc*^Min/+^mice (8 weeks old; 20 ± 2 g, SCXK [SU] 2018–0008) were purchased from GemPharmatech Co., Ltd. (Jiangsu, China). Four mice were housed per cage and were maintained at a constant temperature (23 ± 1°C) and humidity (55 ± 5%) under a 12/12-h light/dark cycle environment. The mice were fed a high-sugar and high-fat diet (D12492; 60% kcal fat, 20% kcal protein and 20% kcal carbohydrate; Xiao Shu You Tai Biotechnology Co., Ltd., Beijing, China) and had ad libitum access to water throughout the course of the study. The mice were adaptively fed for 3 weeks and then randomly divided into the following two groups (*n* = 8 per group): ZQFZ (0.3 g/kg) group and double distilled water (10 mL/kg) group (control), after which they were administered their respective treatment by gavage once a day for another 8 weeks. Body weight was monitored once a week.

### Sample collection and organ index measurement

One hour after the final treatment, blood samples were collected from the caudal vein and the mice were immediately euthanized by CO_2_ inhalation (LY-FL-1; Lingyunboji Technology, Beijing, China). The mice were then immersed in 75% ethanol, and the contents of the cecum were collected under aseptic conditions and stored at −80°C for intestinal microflora analysis. The tissues, including the heart, liver, spleen, lung, kidney, thymus and colorectum were collected, weighed and immediately fixed in 4% paraformaldehyde or stored at −80°C. The above materials were collected for organ index calculations and biochemical and pathological analyses. The organ index was calculated as follows:


$$\text{organ index (mg/g)}=\frac{\text{organ weight (mg)}}{\text{body weight (g)}}$$

### Morphological analysis, histopathological analysis and immunohistochemical examination

The colorectum, with the cecum and anus, was carefully dissected, and the mesenteric adipose tissue was removed. After cleaning the colorectum with PBS, the number of polyps was recorded. The colorectum and organs (heart, liver, spleen, lung and kidney) of three mice in each group were randomly selected and fixed in 4% paraformaldehyde for 24 h. After dehydrating in alcohol, the tissues were embedded in paraffin wax to support thin (< 5 μm) sectioning and were then stained with hematoxylin and eosin, as previously described [22].

The levels of CD4 (bs-0647R, 1:200 dilution), CD8 (bs-0648R, 1:800 dilution; Bioss, Inc., Beijing, China), interferon (IFN)-γ (PA5-95560, 1:1,000 dilution) and IL-4 (PA5-25165, 1:50 dilution; Invitrogen, Carlsbad, CA, USA) in the spleen and colorectal tumors of *Apc*^Min/+^ mice were analyzed by immunohistochemistry, as previously described [23]. The samples were observed under a CKX41 inverted microscope (Olympus, Tokyo, Japan).

### Intestinal microflora analysis

Cecal contents were collected from randomly selected control (*n* = 5) and ZQFZ-treated (0.3 g/kg) mice (*n* = 4) for routine microbiome total DNA extraction and stored at −80°C. The DNeasy PowerSoil Kit (Qiagen, Inc., Hilden, Germany) was used to extract DNA from feces and cecal contents according to the manufacturer’s protocol. A Nanodrop spectrophotometer (ThermoFisher Scientific, Waltham, MA, USA) was used to quantify the DNA, and DNA quality was confirmed by 1.2% agarose gel electrophoresis. The V3-V4 region of the bacterial 16S rRNA genes was amplified using the forward primer 5′-ACTCCTACGGGAGGCAGCA-3′ and the reverse primer 5′-GGACTACHVGGGTWTCTAAT-3′ [24]. The target fragment was amplified by polymerase chain reaction, recovered and purified using VAHTSTM DNA Clean Beads (Vazyme Biotech Co., Ltd., Nanjing, China) and quantified using a PicoGreen dsDNA Assay Kit (Invitrogen, Carlsbad, CA, USA) and a microplate reader (FLx800; BioTek, Winooski, VT, USA). The samples were then used for paired-end sequencing using the MiSeq Reagent Kit v3 (Shanghai Personal Biotechnology Co., Ltd, Shanghai, China) and an Illumina MiSeq instrument (Illumina, San Diego, CA, USA) according to the manufacturer’s protocol. Raw sequence data were demultiplexed using the demux plugin following by primers cutting with cutadapt plugin. Sequences were then merged, quality filtered and dereplicated using functions of fastq_mergepairs, fastq_filter and derep_fullength in Vsearch plugen. All the unique sequences were then clustered at 98% (via cluster_size) followed by chimera removing (via uchime_denovo). At last, the non_chimera sequences were re-clustered at 97% to generate representive sequences and amplicon sequence variants (ASVs) table. The sequences of bacteria were uploaded to the NCBI Sequence Read Archive with accession number PRJNA825157 (https://www.ncbi.nlm.nih.gov/sra/PRJNA825157/). The results were analyzed as previously described [25].

### Isolation of mouse blood cells

Blood samples from three mice in each group were randomly selected for anticoagulant treatment, in which red blood cells were lysed with red blood cell lysis buffer (ThermoFisher Biochemical Products Co., Ltd., Beijing, China). Cells were collected from each sample and adjusted to a concentration of 1 × 10^6^ cells/100 μL. The cells were then stained with fluorescein isothiocyanate (FITC)-conjugated CD3e Monoclonal Antibody (clone 145-2C11, 11-0031-82), Allophycocyanin-conjugated NK1.1 Monoclonal Antibody (clone PK136, 17-5941-82), and Allophycocyanin-conjugated CD28 Monoclonal Antibody (clone 37.51, 17-0281-82) for 30 min at room temperature in the dark to label activated T cells (CD3e^+^CD28^+^) and NK cells (CD3e^-^NK1.1^+^). FITC-conjugated Armenian Hamster IgG Isotype Control (clone eBio299Arm, 11-4888-81), Allophycocyanin-conjugated Mouse IgG2a kappa Isotype Control (clone eBM2a, 17-4724-81) and Allophycocyanin-conjugated Syrian Hamster IgG Isotype Control (17-4914-81, ThermoFisher Biochemical Products Co., Ltd., Beijing, China) were used as isotype controls. A CytoFLEX flow cytometer and CytExpert software (Beckman Coulter, Inc., Indianapolis, IN, USA) were used to determine the percentage of activated T cells and NK cells in the peripheral blood of *Apc*^Min/+^ mice, based on the expression levels of CD3e, CD28 and NK1.1.

### Biochemical analysis

The levels of IgG (FY2057-A), IgM (FY2058-A), IL-1β (FY2040-A), IL-2 (FY2698-A), IL-6 (FY2163-A), IL-8 (FY2123-A), IL-12 (FY2105-A), IFN-γ (FY2182-A), transforming growth factor-β (TGF-β; FY2686-A), triggering receptor expressed on myeloid cells-2 (TREM-2; FY30133-A), TLR4 (FY2816-A), TLR5 (FY30131-A), TLR7 (FY30128-A), granulocyte-macrophage colony-stimulating factor (GM-CSF; FY2185-A; Jiangsu Feiya Biological Technology Co., Ltd., Jiangsu, China) and IL-4 (EK0405; Wuhan Boster Biological Engineering Co., Ltd., Wuhan China) were determined in the serum and colons of *Apc*^Min/+^ mice using enzyme-linked immunosorbent assay kits according to the manufacturer’s instructions.

### Western blotting

ZQFZ-treated cells (Section 2.1.) and tumor tissues collected from *Apc*^Min/+^ mice were lysed with radioimmunoprecipitation assay buffer containing 1% protease inhibitor cocktail (Sigma-Aldrich, St. Louis, MO, USA) and 2% phenylmethanesulfonyl fluoride (Sigma-Aldrich, St. Louis, MO, USA). The protein content of the cell and tissue lysates was measured using a bicinchoninic acid protein assay kit (Merck, Darmstadt, Germany). Equal amounts of protein (40 μg) were separated by 10-12.5% sodium dodecyl sulfate-polyacrylamide gel electrophoresis (Shanghai Epizyme Biomedical Technology Co., Ltd., Shanghai, China) and then blotted onto polyvinylidene fluoride membranes (Merck, Darmstadt, Germany). The membranes were blocked with NcmBlot Blocking Buffer (New Cells and Molecular Biotech Co., Ltd., Suzhou, China) at 4°C for 15 min and then incubated for 16 h at 4°C with the following primary antibodies: anti- inhibitor of NF-κB (IκB)α (A19714), anti-NF-κB (A18210), anti-phosphorylated (P)-NF-κB (Ap0475), anti-P-inhibitor of nuclear factor kappa-B kinase (IKK)α/β (Ap0891; ABclonal, Inc., Woburn, MA, USA), anti-P-IκBα (ab12135; Abcam, Cambridge, UK), anti-glyceraldehyde-3-phosphate dehydrogenase GAPDH (ABS16; Millipore, Merck), anti-IKKα/β (bs-10123R), anti-CD4 (bs-0647R), anti-CD8 (bs-0648R), anti-cytotoxic T-lymphocyte-associated protein 4 (CTLA4, bs-10006R) or anti- programmed death-ligand 1 (PD-L1, bs-4941R, Bioss, Inc., Beijing, China). After washing with Tris-buffered saline containing 0.1% Tween-20, the membranes were exposed to horseradish peroxidase-conjugated secondary antibodies (1:2,000 dilution; E-AB-1001 and E-AB-1003; Elabscience Biotechnology Co., Ltd., Wuhan, China) at room temperature for 2 h. Protein bands were visualized using a Tanon-5200 imaging system (Tanon Science and Technology Co., Ltd., Shanghai, China) with an enhanced chemiluminescence reagent (AR1170, Wuhan Boster Biological Engineering Co., Ltd.). The results were quantified using ImageJ software (National Institutes of Health, Bethesda, MD, USA).

### Statistical analysis

All data are presented as the mean ± standard deviation (SD). Data were analyzed using SPSS 19.0 software (IBM Corporation, Armonk, NY, USA), and a one-way analysis of variance (ANOVA) was used to detect significance differences between groups. A *p* value < 0.05 was considered to be statistically significant.

### Ethics statement

The animal study was reviewed and approved by the Animal Ethics Committee of Jilin University.

## RESULTS

### Effect of ZQFZ on the inhibition of the NF-κB pathway by LPS in RAW264.7 cells

The protein levels of IKKα/β, IκBα and NF-κB were determined in ZQFZ-treated LPS-stimulated RAW264.7 cells to determine whether ZQFZ regulates the NF-κB signaling pathway. As shown in Supplementary Figure 1, the phosphorylation levels of IKKα/β, IκBα and NF-κB increased after LPS treatment. The 0.25 mg/mL and 0.5 mg/mL ZQFZ treatments significantly decreased the P-IKKα/β/total IKKα/β ratio by 24.85% (*p* < 0.001) and 50.70% (*p* < 0.001), respectively; the P-IκBα/total IκBα ratio by 36.97% (*p* < 0.001) and 33.39% (*p* < 0.05), respectively; and the P-NF-κB/total NF-κB ratio by 88.53% (*p* < 0.001) and 55.92% (*p* < 0.001), respectively, compared with the corresponding ratios in the untreated cells.

### Inhibitory effect of ZQFZ on CRC in Apc^Min/+^ mice

**Figure 1:**
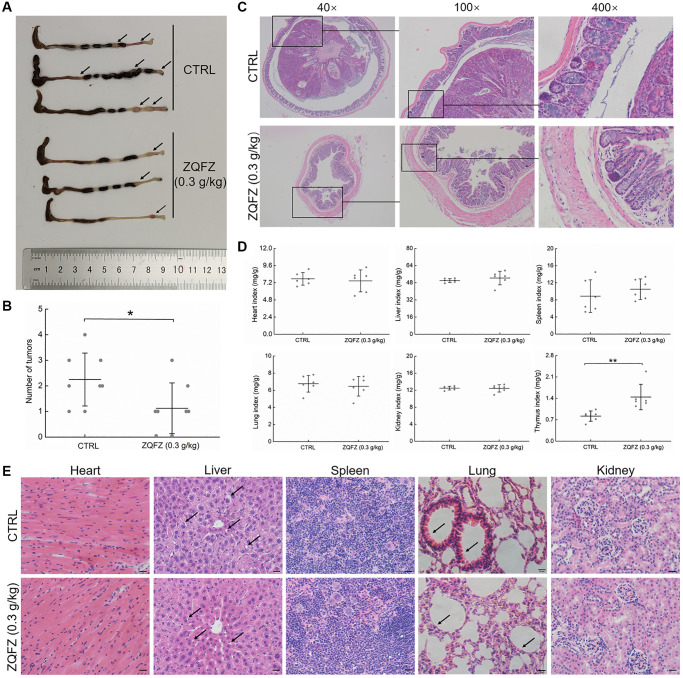
**The improving effect of ZQFZ on colorectum and viscus in *Apc*^Min/+^ mice.** (**A**) The effect of ZQFZ on the colorectal morphology, black arrows indicated colorectal tumors. (**B**) The effects of ZQFZ on the number of colorectal tumors (*n* = 8 mice/group). H&E staining was used to evaluate pathological alterations of (**C**) colorectum (400×, scale bar: 20 μm), (**D**) The effects of ZQFZ on organ indexes (*n* = 6 mice/group), (**E**) heart, liver, spleen, lung and kidney (400×, scale bar: 20 μm) under a light-microscope digital camera (*n* = 3 mice/group), black arrows indicated with vacuolar degeneration in the liver and inflammatory cell infiltration in pulmonary alveoli. Data was shown as the mean ± SD and determined via a one-way ANOVA test. ^**^p < 0.01 vs. CTRL group.

In *Apc*^Min/+^ mice, ZQFZ (0.3 g/kg) administration markedly decreased the size (Figure 1A) and the number of tumors (Figure 1B) in colorectal tissue, inhibited intestinal hyperplasia and restored normal intestinal villi structure (Figure 1C). ZQFZ (0.3 g/kg) administration increased the body weight of the mice, but only after 56 days (*p* < 0.05, Supplementary Figure 2).

ZQFZ reduced the incidence of vacuolar degeneration in the liver and decreased inflammatory cell infiltration in the incrassated alveolar diaphragm (Figure 1E). ZQFZ increased the thymus index value (*p* < 0.01), but had no significant effect on the structure (Figure 1E) or index values (Figure 1D) of other organs, including the heart, liver, spleen, lung and kidney, which indicated its safety.

### The regulatory effect of ZQFZ on the intestinal microflora of Apc^Min/+^ mice

**Figure 2:**
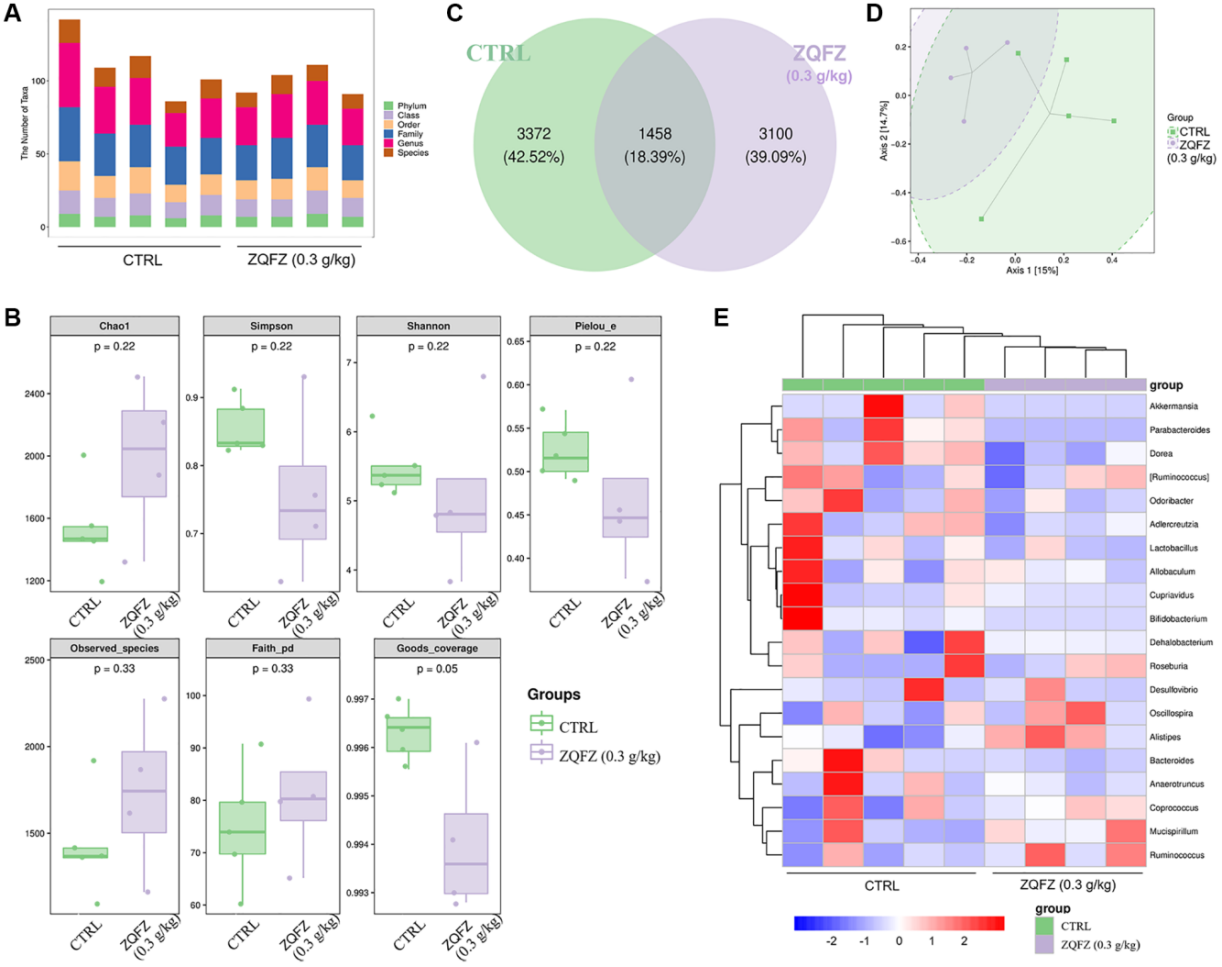
**ZQFZ regulated the intestinal microflora.** (**A**) Statistical chart of microbial taxon (**B**) Chao1, Simpson, Shannon, Pielou’s evenness, observed species, Faith’s phylogenetic diversity and Good’s coverage index values from alpha diversity analysis of the two groups. (**C**) Venn diagram. (**D**) PCoA of beta diversity analysis. (**E**) Heatmap of the 20 bacterial genera with the most significantly different abundance, clustered for UPGMA according to euclidean distance of species composition data. Data are expressed as the mean (*n* = 4 or 5 mice/group).

Compared with control mice, ZQFZ-treated mice had an altered composition of the microbial colonies in the intestine, according to the diversity of the composition spectrum of the microbial community (Figure 2A and 2D). Alpha diversity reflects the diversity of microbial communities based on a variety of indicators, including the Chao1 and observed species indices (characterization of abundance), the Shannon and Simpson index (characterization of diversity), Faith’s phylogenetic diversity index (characterization of diversity based on evolution), Pielou’s evenness index (characterization of evenness) and Good’s coverage index (characterization of coverage) [26]. The richness and diversity based on evolution index values showed upward trends, but the diversity, evenness and coverage index values showed downward trends in the ZQFZ-treated mice compared with the control mice, based on the grouped box plot of the alpha diversity index values. However, no significant differences were observed between the groups (Figure 2B). Meanwhile, of the 7,930 ASVs detected, 1,458 (18.39%) were found in both control and ZQFZ-treated mice. In ZQFZ-treated mice, 3,100 unique ASVs (39.09%) were detected and in control mice, 3,372 (42.52%) were detected, indicating a difference in bacterial community composition between the two groups (Figure 2C). The bi-clustered genus-level species composition heat map analysis showed that the top 20 nodes in terms of richness in the intestine were altered after ZQFZ treatment (Figure 2E). According to the linear discriminant analysis (LDA) effect size, 11 nodes were significantly changed by ZQFZ treatment (*p* < 0.05, LDA > 2), such as Porphyromonadaceae (family), Mogibacteriaceae (family), Verrucomicrobia (phylum) and Actinobacteria (class) (Table 1).

**Table 1:** The dominant nodes based on LEfSe analysis of intestinal microflora in mice.

**Taxa**	**Group**	**Abundance**	**LDA_score**	***P* value**
Bacteria.Actinobacteria.Actinobacteria	CTRL	3.2250	3.589	0.0105
Bacteria.Actinobacteria.Actinobacteria.Bifidobacteriales	CTRL	3.2096	3.621	0.0105
Bacteria.Actinobacteria.Actinobacteria.Bifidobacteriales.Bifidobacteriaceae.Bifidobacterium	CTRL	3.2073	3.574	0.0105
Bacteria.Bacteroidetes.Bacteroidia.Bacteroidales.Porphyromonadaceae	CTRL	3.6871	3.510	0.0105
Bacteria.Bacteroidetes.Bacteroidia.Bacteroidales.Porphyromonadaceae.Parabacteroides	CTRL	3.6871	3.510	0.0105
Bacteria.Firmicutes.Clostridia.Clostridiales._Mogibacteriaceae	CTRL	3.8920	3.757	0.0143
Bacteria.Verrucomicrobia	CTRL	4.5817	4.299	0.0143
Bacteria.Verrucomicrobia.Verrucomicrobiae	CTRL	4.5816	4.230	0.0143
Bacteria.Verrucomicrobia.Verrucomicrobiae.Verrucomicrobiales	CTRL	4.5816	4.229	0.0143
Bacteria.Verrucomicrobia.Verrucomicrobiae.Verrucomicrobiales.Verrucomicrobiaceae	CTRL	4.5816	4.229	0.0143
Bacteria.Verrucomicrobia.Verrucomicrobiae.Verrucomicrobiales.Verrucomicrobiaceae.Akkermansia	CTRL	4.5816	4.230	0.0143

Data are presented as the mean and analyzed via a Wilcoxon test (*n* = 4 or 5 mice/group). The advantage group, logarithm abundance of dominant nodes, LDA score and *P* value were provided.

### The immunomodulatory effect of ZQFZ in Apc^Min/+^ mice

**Figure 3:**
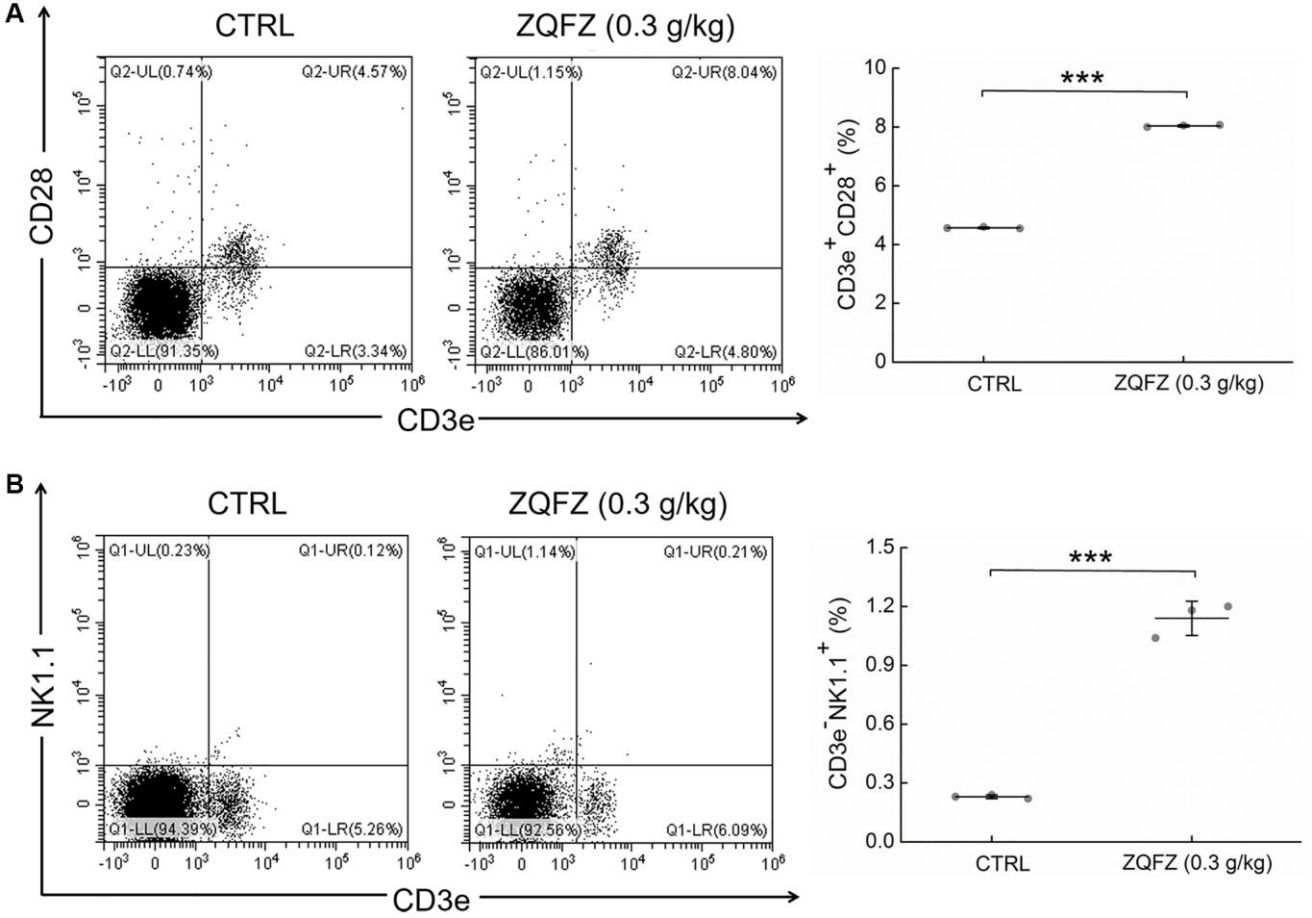
**ZQFZ enhanced the levels of immune cells component.** Analyzed by flow cytometry, ZQFZ enhanced (**A**) the levels of T cells (represented by CD3e^+^CD28^+^) and (**B**) the levels of NK cells (represented by CD3e^-^NK1.1^+^) in peripheral blood of *Apc*^Min/+^ mice. Data was shown as the mean ± SD (*n* = 3 mice/group) and determined via a one-way ANOVA test. ^***^p < 0.001 vs. CTRL group.

Compared with control mice, ZQFZ-treated mice had significantly increased proportions of CD3^+^CD28^+^ T cells (4.57% vs. 8.04%, *p* < 0.001, Figure 3A) and NK cells (0.23% vs. 1.14%, *p* < 0.001, Figure 3B) in the peripheral blood.

**Figure 4:**
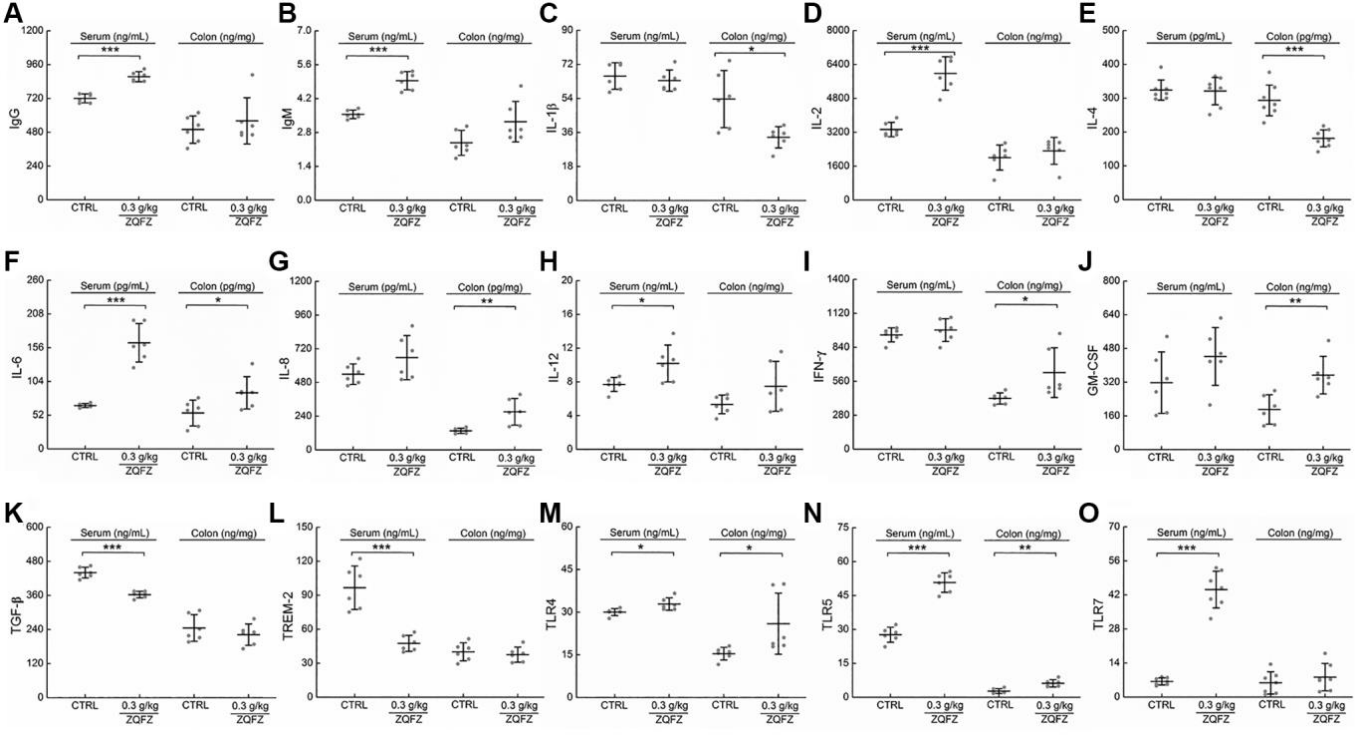
**ZQFZ regulated the cytokines related to immunity.** The levels of 15 factors related to immunity in the serum and colorectum of *Apc*^Min/+^ mice were detected by ELISA kits: (**A**) IgG, (**B**) IgM, (**C**) IL-1β, (**D**) IL-2, (**E**) IL-4, (**F**) IL-6, (**G**) IL-8, (**H**) IL-12, (**I**) IFN-γ, (**J**) GM-CSF, (**K**) TGF-β, (**L**) TREM-2, (**M**) TLR4, (**N**) TLR5 and (**O**) TLR7. The data was expressed as the mean ± SD and analyzed via a one-way ANOVA test (*n* = 6 or 7 mice/group). ^*^p < 0.05, ^**^p < 0.01 and ^***^p < 0.001 vs. CTRL group.

Changes in an ecological imbalance of the intestinal microbiota lead to abnormal immune responses and thus increase the risk of CRC [27]. Flow cytometry results showed that ZQFZ had a regulatory effect on immune cells in the peripheral blood of *Apc*^Min/+^ mice. Therefore, we analyzed cytokine levels in the serum and tumors of *Apc*^Min/+^ mice. ZQFZ significantly increased the serum levels of IgG (*p* < 0.001, Figure 4A), IgM (*p* < 0.001, Figure 4B), IL-2 (*p* < 0.001, Figure 4D), IL-12 (*p* < 0.05, Figure 4H) and TLR7 (*p* < 0.001, Figure 4O) and decreased the levels of TGF-β (*p* < 0.001, Figure 4K) and TREM-2 (*p* < 0.001, Figure 4L). Concurrently, ZQFZ increased the levels of IL-8 (*p* < 0.01, Figure 4G), IFN-γ (*p* < 0.05, Figure 4I) and GM-CSF (*p* < 0.01, Figure 4J) and decreased the levels of IL-1β (*p* < 0.05, Figure 4C) and IL-4 (*p* < 0.001, Figure 4E) specifically in the colon. The levels of IL-6 (*p* < 0.05, Figure 4F), TLR4 (*p* < 0.05, Figure 4M) and TLR5 (*p* < 0.01, Figure 4N) were upregulated by ZQFZ treatment in both the serum and colon.

**Figure 5:**
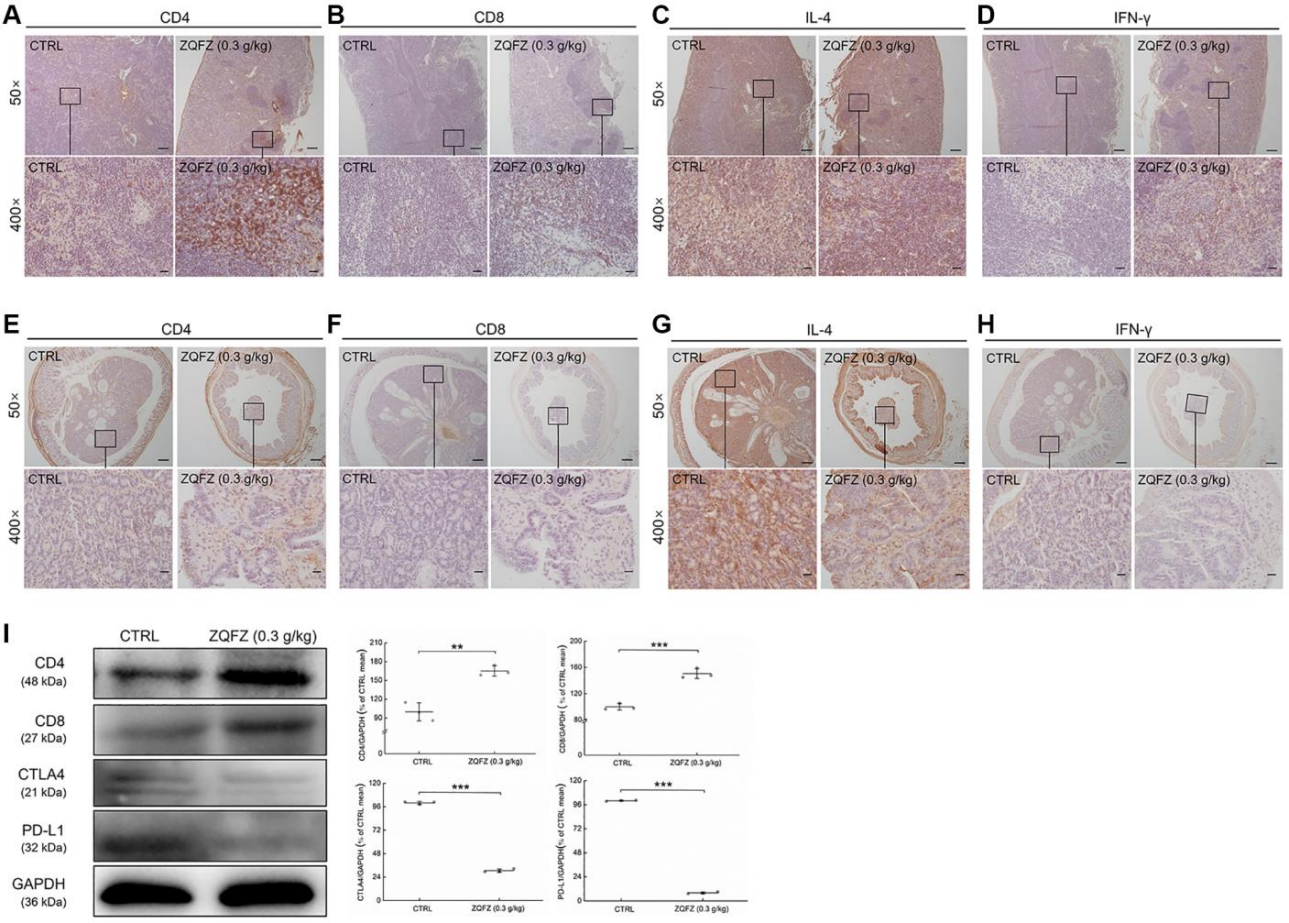
**ZQFZ regulated immune-related proteins of spleen and colorectum in *Apc*^Min/+^ mice.** Base on the result of immunohistochemical staining, ZQFZ enhanced (**A**) CD4 and (**B**) CD8, no affect (**C**) IL-4, and enhanced (**D**) IFN-γ in spleen, and enhanced (**E**) CD4 and (**F**) CD8, no affect (**G**) IL-4, and enhanced (**H**) IFN-γ in colorectal tumors (50×, scale bar: 200 μm; 400×, scale bar: 20 μm). The protein expression levels of (**I**) CD4, CD8, CTLA4 and PD-L1 in colorectal tumor were detected by western blotting. The quantitative data of the protein expression levels were normalized by GAPDH expressions and were shown as a percentage of the corresponding relative intensity of the CTRL group. Data are shown as the mean ± SD and analyzed via a one-way ANOVA test. (*n* = 3). ^**^p < 0.01 and ^***^p < 0.001 vs. CTRL group.

CD4^+^ and CD8^+^ T cells are the main indicators of cellular immune responses [28]. CD4^+^ T cells further differentiate into Th1 and Th2 cells. IFN-γ and IL-4 are the characteristic cytokines of Th1 and Th2 cells, respectively, and they are used to determine the number of cells [29, 30]. According to the images of immunohistochemically stained sections, ZQFZ increased the number of CD4^+^ and CD8^+^ T cells (Figure 5A, 5B, 5E, 5F), and IFN-γ levels in both colorectal tumors and the spleen (Figure 5D, 5H), but had no effect on IL-4 levels (Figure 5C, 5G). Western blotting analysis of the colorectal tumors confirmed that treatment with 0.3 g/kg of ZQFZ significantly upregulated the expression levels of CD4 by 65.16% (*p* < 0.01) and of CD8 by 50.63% (*p* < 0.001), compared with their levels in control mice. In contrast, the expression levels of CTLA4 and PD-L1 were significantly decreased by 69.38% (*p* < 0.001) and 92.01% (*p* < 0.001), respectively, after ZQFZ treatment (Figure 5I).

## DISCUSSION

In this study, the inhibition of CRC growth by ZQFZ was demonstrated in *Apc*^Min/+^ mice. This effect may have been achieved by the regulation of the abundance of specific intestinal microbes and by increasing the immune response. To the best of our knowledge, our study is the first to document the anti-CRC effects of ZQFZ. Treatment with *Astragalus membranaceus* polysaccharides can inhibit the expression of PD-L1 [31], and part of couplet medicines of *Astragalus membranaceus* showed the CRC inhibition effect [32, 33]. However, the anti-CRC effect of ZQFZ based on immune response regulation has not been systematically studied. In a previous study, we showed that ZQFZ increases the activity of NK cells in immunosuppressed mice [21]. NK cells have anti-tumor effects by releasing perforin, cytoplasmic granules and granzyme; inducing death-receptor-dependent apoptosis and mediating antibody-dependent cytotoxicity [34]. The NF-κB signaling pathway is involved in regulating cell proliferation and apoptosis. As previously reported, NF-κB inhibitor treatment significantly increases the chemosensitivity of the colon cancer cell line HT-29, and it effectively inhibits cell proliferation and induces apoptosis when used in combination with chemotherapeutic drugs [35]. *In vitro* studies showed that ZQFZ suppressed the phosphorylation-mediated activation of IKKα/β/IκBα/NF-κB signaling in LPS-treated RAW264.7 cells. This suggests that ZQFZ has potential cancer inhibitory effects.

ZQFZ treatment inhibited the development of colorectal tumors and intestinal hyperplasia in *Apc*^Min/+^ mice. The intestine is the largest digestive organ of the body and is also one of the largest organs in contact with the external environment. The intestinal microflora plays an important role in maintaining intestinal homeostasis, and the appropriate stimulation of specific microbes promotes the maturation of intestinal mucosa-related lymphoid tissue [36]. Inappropriate microbial stimulation leads to impaired intestinal immune function, leading to an increased incidence of certain intestinal diseases, including ulcerative colitis, Crohn’s disease and CRC [37, 38]. Changes in the intestinal microflora may affect the homeostasis of the immune microenvironment in the intestinal region and thus affect the efficacy of disease treatment. For instance, secondary metabolites from Actinobacteria have immunosuppressive activity by suppressing cytokine expression and T cells proliferation [39, 40]. Verrucomicrobia are common in the colonic microbiota, and their abundance has been shown to positively correlate with TNF-α levels in patients with celiac disease [41]. ZQFZ treatment significantly regulated the abundance of Actinobacteria and Verrucomicrobia in the intestinal microflora of *Apc*^Min/+^ mice. These results indicated that ZQFZ treatment directly changed the abundance of certain immune-associated functional microbes or regulated the immune system to inhibit tumor growth. The immune system plays various roles in the occurrence and development of cancer [42], Cancer immune surveillance has been proposed for more than 50 years [43]. Increased infiltration of CD8^+^ T cells is associated with decreased tumor recurrence and improved patient survival [44–46]. To escape CD8^+^ T cells reactivity, major histocompatibility complex (MHC) class I proteins are aberrantly expressed in primary tumors [47, 48]. These tumor cells then become potential targets of NK cells, as NK cells recognize and kill cells with low levels of or absent MHC class I expression [49]. Thus, the contribution of NK cells to immune surveillance in CRC has become a topic of research focus [50]. Flow cytometry showed that ZQFZ treatment increased the number of CD3e^+^CD28^+^ cells and CD3e^-^NK1.1^+^ cells in the peripheral blood of *Apc*^Min/+^ mice, indicating that the anti-CRC effect of ZQFZ may be achieved by increasing the number of immune cells. ZQFZ treatment also significantly affected the expression levels of multiple immune-related cytokines. As an activating receptor of the Ig superfamily, TREM2 acts as an amplifier of the immune response [51]. IL-12 and IL-2 promote T cells proliferation and IFN-γ expression by stimulating mitogens or CD3. The anti-cancer effect of T cells is greatly enhanced when IL-12 and IL-2 are stimulated simultaneously [52]. IL-2 also promotes the survival, proliferation and cytotoxicity of NK cells [53, 54]. TLRs connect the activation of the innate immune system and the adaptive immune system and play important roles in anti-cancer immunity [55]. TLR4 has a tumor-promoting effect [56]; however, monophosphoryl lipid A, a TLR4 agonist, enhances the adaptive immune response by promoting Th1 cell differentiation and increasing IFN-γ expression levels [57]. TLR5 and TLR7 have significant anti-tumor effects through dendritic cell-mediated cytotoxic T cells activation and regulatory T cell (Treg) inhibition [58]. In a therapeutic cancer vaccine model, flagellin was shown to improve the response of tumor-specific CD8^+^ T cells after TLR5 stimulation [59]. Treatment with the TLR7 agonist imiquimod promotes the infiltration of CD4^+^ and CD8^+^ T cells, decreases the absolute number of Tregs in the tumor microenvironment and establishes anti-tumor immune memory in mice [60, 61]. Our cytokine analysis and flow cytometry results further confirmed that the anti-CRC effect of ZQFZ was related to immune regulation.

CD8^+^ cytotoxic T cells are considered to be the basis of the anti-cancer immune response [62]. CD4^+^ Th1 cells secrete IFN-γ and chemokines to promote the expansion and initiation of CD8^+^ T cells and their infiltration to the tumor site [63]. IL-4 is the main product of CD4^+^ Th2 cells and is also the key factor in the development of Th2 cells [64]. IL-4 promotes the production of H_2_O_2_, which induces DNA damage in malignant gastrointestinal tumors [65]. CTLA4 and programmed cell death protein 1 (PD-1) expressed on the surface of T cells, act as inhibitory receptors and block the immune function of T cells using different mechanisms [66, 67]. CTLA-4 binds to B7 protein on activated antigen-presenting cells to reduce the T-cell immune response, and PD-1 inhibits T cell activity by binding to two B7 family members, PD-L1 and PD-L2 [66, 67]. After treatment with ZQFZ, the proportion of CD4- and CD8-positive cells in the spleen and colorectal tumors of *Apc*^Min/+^ mice increased significantly and the proportions of IFN-γ- and IL-4-expressing cells were also altered. Concurrently, the protein levels of CD4 and CD8 in colorectal tumors of *Apc*^Min/+^ mice also increased significantly after ZQFZ treatment. Meanwhile, we demonstrated that ZQFZ decreased the protein levels of CTLA4 and PD-L1 in colorectal tumors of *Apc*^Min/+^ mice. These results suggested that ZQFZ exerted anti-CRC effects at least partly by regulating the activation of immune cells.

## CONCLUSIONS

This study demonstrated that ZQFZ inhibits the development of CRC in *Apc*^Min/+^ mice by regulating the intestinal microflora and the immune response associated with the activation of T cells. These data provide a basis for the use of ZQFZ in the treatment of CRC in the clinic.

## Supplementary Materials

Supplementary Figures
