# Supplementary material for: ZhenQi FuZheng formula inhibits the growth of colorectal tumors by modulating intestinal microflora-mediated immune function
Source: Aging (Albany NY). 2022 Jun 8;14(11):4769–85. doi: 10.18632/aging.204111 (PMC9217701; doi:10.18632/aging.204111)
Supplement: Supplementary Figures [file aging-14-204111-s001.pdf]

# SUPPLEMENTARY FIGURES

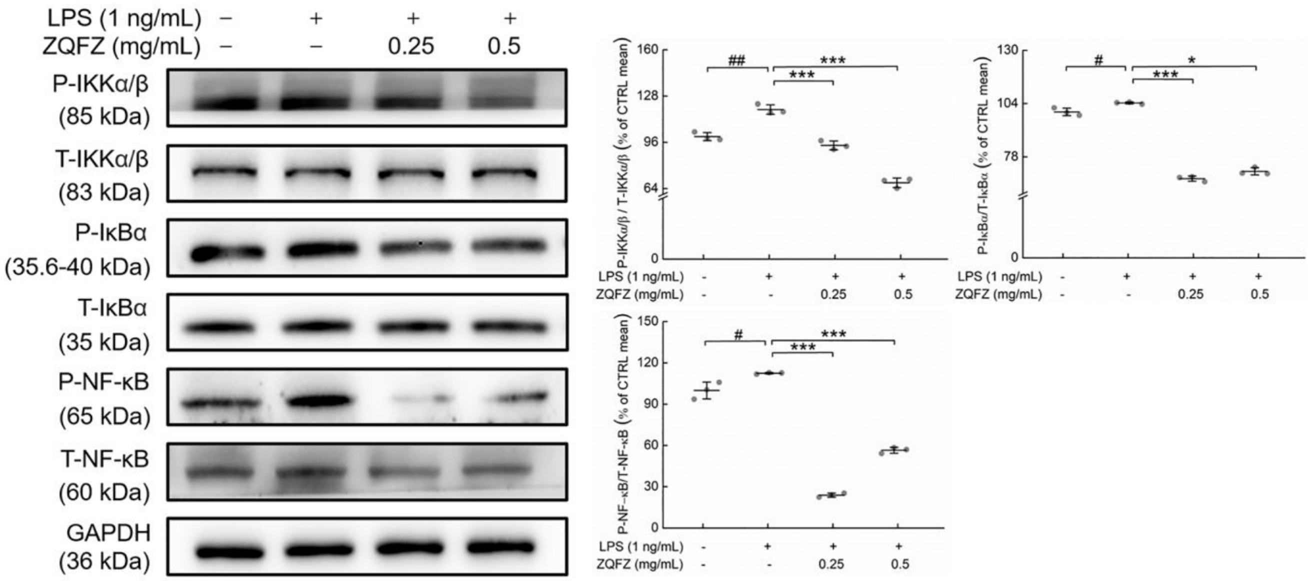


**Supplementary Figure 1. The expression levels of P-IKKα/β, P-IκBα and P-NF-κB in RAW264.7cells were detected by Western blotting.** The quantitative data of the protein expression levels were normalized by related total protein expressions. Data are shown as the mean ± SD and analyzed via a one-way ANOVA test. (*n* = 3). $^{\#}p < 0.05$ and $^{\#\#}p < 0.01$ vs. control group, $^{*}p < 0.05$ and $^{***}p < 0.001$ vs. model group.

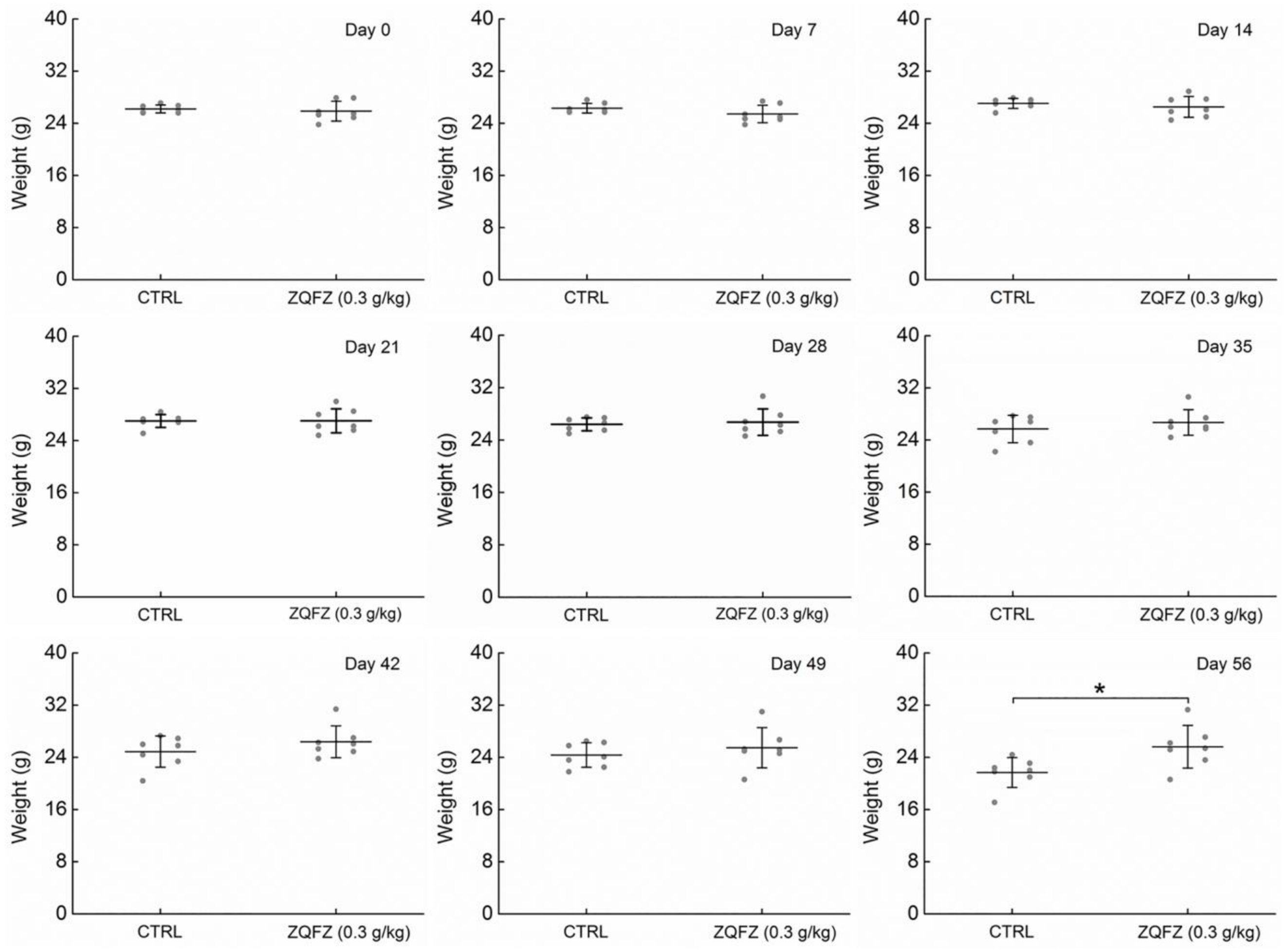


**Supplementary Figure 2. The effects of ZQFZ on bodyweight of *Apc*$^{Min/+}$ mice.** Data are shown as the mean ± SD and analyzed via a one-way ANOVA test. (*n* = 7 mice/group). $^{*}p < 0.05$ vs. control group.
